# Pathophysiologic consequences following inhibition of a CFTR-dependent developmental cascade in the lung

**DOI:** 10.1186/1471-213X-5-2

**Published:** 2005-02-04

**Authors:** J Craig Cohen, Janet E Larson

**Affiliations:** 1 Louisiana State University Health Sciences Center, Departments of Medicine, Biochemistry, and Genetics, School of Medicine, New Orleans, LA 70112, USA; 2 Ochsner Children's Research Institute, Ochsner Clinic Foundation, New Orleans, LA 70121, USA

## Abstract

**Background:**

Examination of late gestation developmental genes *in vivo *may be limited by early embryonic lethality and compensatory mechanisms. This problem is particularly apparent in evaluating the developmental role of the cystic fibrosis transmembrane conductance regulator (CFTR) gene in the cystic fibrosis (CF) phenotype. A previously described transient *in utero *knockout (TIUKO) technology was used to address the developmental role of CFTR in the rat lung.

**Results:**

Rat fetuses transiently treated with antisense *cftr in utero *developed pathology that replicated aspects of the human CF phenotype. The TIUKO CF rat developed lung fibrosis, chronic inflammation, reactive airway disease, and the CF Antigen (MRP8/14), a marker for CF in human patients, was expressed.

**Conclusions:**

The transient *in utero *antisense technology can be used to evaluate genes that exhibit either early lethality or compensating gene phenotypes. In the lung CFTR is part of a developmental cascade for normal secretory cell differentiation. Absence of CFTR results in a constitutive inflammatory process that is involved in some aspects of CF pathophysiology.

## Background

The *in utero *gene transfer technology devised in this laboratory [1] was originally developed to circumvent the inflammatory response seen after birth with adenoviral-mediated gene transfer. During the course of these experiments it was discovered that the *in utero *transfer of the gene for cystic fibrosis transmembrane conductance regulator (*cftr*) to normal rat fetuses resulted in phenotypic changes in the neonatal lungs [2]. At the time of gene transfer the targeted epithelial cells were undifferentiated multipotential cells [3]. Administration of *cftr *to this epithelium using an adenovirus vector system resulted in persistent phenotypic changes in cells although the expression of the transgene was transient. These data provided the first insight that CFTR expression during the fetal period could permanently alter the differentiation of lung epithelial cells. The permanent functional changes in the *in utero cftr*-treated rats included an enhanced resistance to pulmonary bacterial infection three months after birth [2].

At the same time, other laboratories were examining the temporal and tissue-specific expression of CFTR. CFTR lung expression is greatest during the fetal period where it is localized to airway epithelial undifferentiated multipotential cells [4-9]. As these multipotential cells differentiate, the expression of CFTR dissipates and the adult lung expresses only a fraction of that expressed during the fetal period. Thus, CFTR resembles other developmentally important genes in its expression at specific times during organogenesis [10-12].

In addition to its role as a chloride channel in the mature lung, CFTR's expression in undifferentiated epithelial cells suggested another role (or roles) during development. Moreover, this raised the question of how much of CF disease pathology could be attributed specifically to the lack of CFTR expression during differentiation and how much could be attributed to lack of a chloride channel in the mature lung.

These questions prompted further experiments by this laboratory in the CF knockout mouse. Reversal of the lethal phenotype of the CF (*cftr *-/-) mouse following transient *in utero *expression of *cftr *[13] confirmed the role of this gene in gut development. Because of the rapid cell turnover, the human CFTR transgene was detected in the fetal gut for up to 72 hours post-treatment but not after birth. The *in utero *gene therapy did not permanently replace the CFTR-encoded cAMP-dependent chloride channel but rescued the mice from the disease phenotype and reversed biochemical markers specific to the knockout phenotype [14]. These data established that extra uterine expression of CFTR was not required for the correction of the intestinal obstruction in *cftr *-/- mice.

Additional insight into the role of CFTR in secretory cell development was obtained when we began to examine the effects of CFTR following *in utero *over expression in homozygous normal mouse pups and discovered that over expression resulted in a lethal phenotype due to epithelial cell hyperplasia [11]. Characterization of the secretory epithelium following CFTR over expression during fetal development has now demonstrated accelerated lung epithelial cell differentiation in the rat, mouse and nonhuman primate [2,14-16].

Experiments examining the developmental role of CFTR relied on either a knockout mouse model that poorly mimicked human lung disease or over expression studies in normal animals. At this point it seemed that only two research approaches were available to determine which aspects of CF pathology were due to the lack of CFTR expression during development. Reversal of human CF by transient *in utero *gene therapy is currently under consideration by our laboratory, but is many years from practical therapeutic consideration. Alternately, one could attempt to transiently inhibit in animals models CFTR production *in utero *to induce aspects of the adult CF disease phenotype in the presence of normal adult levels of CFTR.

The *in utero *gene transfer method developed by this laboratory uses small quantities of recombinant adenovirus at times during gestation when the lung and intestine epithelium is largely composed of undifferentiated multipotential cells [1]. Recombinant adenoviruses at 10^8 ^pfu/ml of amniotic fluid have been transferred to mice, rats, and rhesus monkeys [2,14-16]. The high transfer efficiency and absent immune response suggested that it was possible to use recombinant adenoviruses to transfer antisense genes to the lung and intestinal epithelium and transiently inhibit gene expression. Because undifferentiated multipotential cells were targeted, transfer of the antisense analog of a developmentally active gene would have the potential to significantly affect the developmental cascades in the lung and intestines. Recently, we developed a transient *in utero *knockout (TIUKO) technology to inhibit expression of specific genes in the fetal lung and intestine [17]. In this paper the TIUKO technology is applied to the question of the developmental role of CFTR in the cystic fibrosis phenotype.

## Results

### Inhibition of CFTR expression using TIUKO

An adenovirus was constructed from a ATCC plasmid containing exons 1–6 of *cftr *included in the [18] cloned into a recombinant adenovirus in the 3'-5', antisense direction (AdCMVAScftr). This virus was used for *in utero *gene transfer into fetal rats at 16 days gestation.

Sprague-Dawley rats were used in these experiments. There were 75 rats from 7 litters in the control group and 114 rats from 11 litters in the TIUKO group. The rats were treated with AdCMVAScftr at 16 days gestation and were evaluated daily from 18–22 days gestation and up to 1 year following birth.

The choice of controls for these experiments was a primary consideration. We showed in several publications that over expression of CFTR in normal mice and rats results in altered lung morphology. Thus, neither *cftr *constructs nor any sense portion of this gene could be used as a control. An adenovirus with exons 1–6 in the sense direction would not express a truncated product and thus its expression could not be detected as a control. The adenovirus constructs with beta-galactosidase (AdCMVlacZ) and green fluorescent protein (AdCMVgfp) were used in previous experiments in several hundred individual fetuses with no effect on the viability, structure, or function of the lung [1,15,16]. Thus, these two adenoviruses with reporter genes were used as negative controls for normal organogenesis.

**Figure 1:**
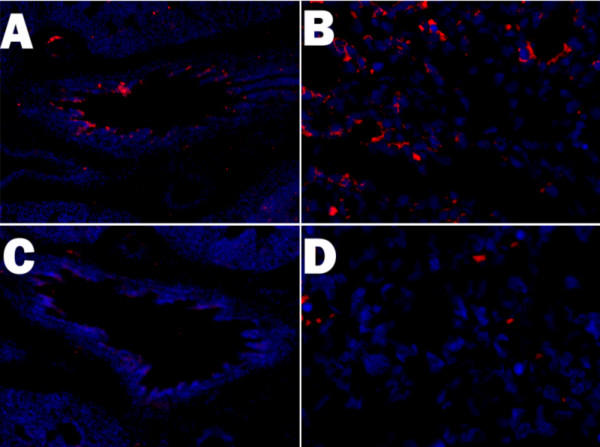
**Inhibition of CFTR expression in rats treated *in utero *with antisense c *ftr ***. Sprague-Dawley rats at 16 days of age were treated with either AdCMVlacZ (Panel A, B, C) or AdCMVAScftr (Panel D). At 19 days gestation lungs were harvested and CFTR expressing cells visualized by fluorescent microscopy with an Alexa 568 (RED) secondary and a goat anti-CFTR primary antibody. Nuclei were stained with DAPI (BLUE). Original Magnification Panels A & C 100×; Panels B & D 400×.

The effects of AdCMVAScftr on CFTR expression in rat tissues was compared to control (AdCMVlacZ-treated) lungs at 24 and 72 hours post antisense therapy by fluorescent immunohistochemistry. As shown in Fig. 1A, deconvolution microscopic analysis readily detected CFTR in the normal embryonic lungs. The specificity of the immunohistochemistry was shown by the blocking of all fluorescence using specific blocking peptides (Fig. 1C). Comparison of control (Fig. 1B) and antisense CFTR treated (Fig. 1D) revealed decreased expression of CFTR in antisense treated lungs.

**Figure 2:**
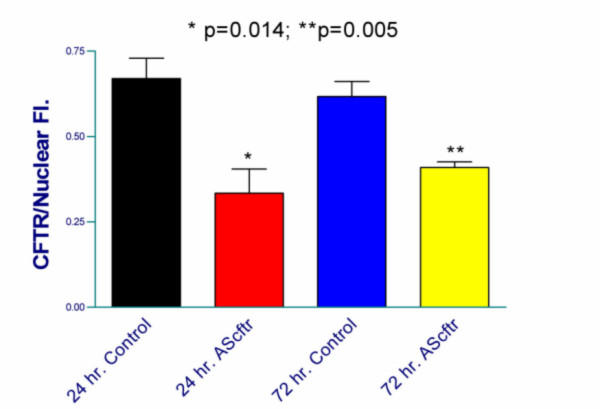
**Quantitation of CFTR expression following infection with AdCMVAScftr**. Fetuses were treated at 16 days gestation with either AdCMVlacZ (control) or AdCMVAScftr and lungs harvest at 24 and 72 hours post-gene transfer. Lungs from 5 animals were cryo-sectioned and CFTR visualized by immunohistochemistry. Image analysis on the deconvoluting microscope was performed and the results standardized for the number of cells by nuclear staining with DAPI

Reduction of the antisense transgene expression is difficult to measure, because at a maximum only 10^8 ^cells would be affected if one achieved 100% infection efficiency. Transfection efficiency via the amniotic fluid is less than 10% so only between 10^6–7 ^cells are affected in the tissue. Thus, real time PCR, northern blots, and western blots lack the sensitivity to detect these changes as shown in our previous publication on the TIUKO *c-myc *mouse [17]. Thus, the only method available to quantitate reduction in target gene expression in the TIUKO method is image analysis of random sections from multiple, independently treated lungs. As shown in Fig. 2, image analyses of the relative levels of CFTR expressed per cell in the AdCMVAScftr-treated tissues were performed.

Statistically significant reduction in CFTR levels was observed at 24 (p = 0.014) and 72 hours (p = 0.005) post-TIUKO *cftr *therapy. Thus, as in our previously published TIUKO *c-myc *model, the TIUKO *cftr *therapy decreases expression of the target gene at a critical time in lung development.

### Airway and parenchymal changes in the CF TIUKO rat

Focal areas of fibrosis are seen in the lungs of congenic CF mice as well as humans at autopsy [19]. Rats were followed sequentially for lung histological examination to determine if they would develop any chronic lung changes that mimicked CF lung pathology. Both the airways and parenchyma were examined.

Comparison of the histology of lungs from control and AdCMVAScftr-treated animals during the neonatal period revealed little or no gross structural pathology (data not shown). Development of pulmonary histopathology became apparent in adult rats by 100 days of age following the *in utero *antisense *cftr *treatment.

The most notable histologic change was in the airways, which appeared thickened and fibrosis. Morphometric analysis was used to quantitate airway wall dimensions on lung sections from 100 day old rats following staining with hematoxylin and eosin [20,21]. The wall area was determined by digitizing the area excluding airway epithelium and cartilage. The corresponding segment of sub epithelial basement membrane was digitized and used as a reference length to normalize airway wall area[21]. As shown in Table 1, there was a significant increase in airway wall thickness in the *in utero *antisense *cftr *treated animals as compared to their aged-matched controls. The average internal airway circumference was not statistically different between the treated and control group. These measurements insured that similar sized airways were compared between the two groups.

**Table 1:** Morphometric analysis of fibrosis and airway thickness in control and AdCMVAScftr treated lungs at 100 days of age

**Sample**	**Average Internal Circumference (I) N = 20/group**	**Average area (A)**	**Average A/I**	**Average airway Protein (V) N = 24/group**	**average collagen (C)**	**average C/V**
AdCMVgfp-treated	101.8	452.1	4.49	6595 ± 1829	2921 ± 810	0.5251
AdCMVAScftr-treated	114.7	833.9	7.27*	6965 ± 911	11665 ± 1829	1.6648**

* p = .023 ** p < 0.001

**Figure 3:**
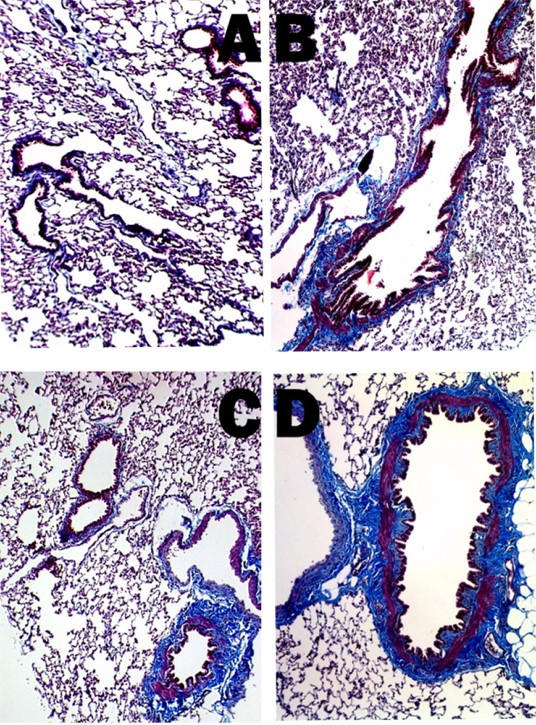
**Airway fibrosis following *in utero *AdCMVAScftr therapy**. Lung sections were stained with Masson's Trichrome to visualize collagen (blue) in 100 day old rats. There was increased collagen in both the small (Panel C) and large airways (Panel D) of the AdCMVAScftr-treated rats when compared to control airways of the same size (Panels A and B). The morphometric quantitation of this fibrosis (Table 1) confirmed that these changes were consistent throughout the lung fields examined. Original magnifications 100×.

**Figure 4:**
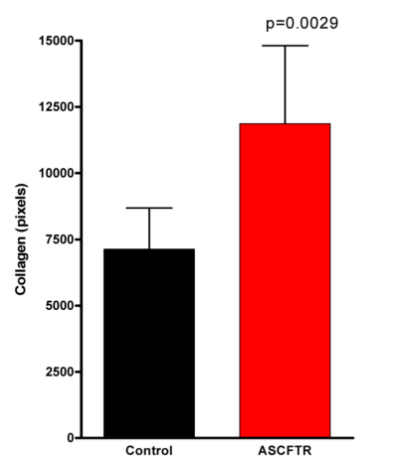
**Collagen in rat lungs following *in utero *antisense *cftr *gene transfer**. Rat fetuses at 16 days gestation were treated with either AdCMVlacZ (control; black) or AdCMVAScftr (ASCFTR; red). At 120 days of age 5 animals in each group were harvested and 5 random sections were analyzed for total collagen following Mason trichome staining using image analysis as described in Methods

Masson's Trichrome was used to differentiate collagen from smooth muscle and elastin surrounding the airways to better visualize and quantitate the extent of airway fibrosis. Collagen following this stain was visualized as a dense bluish-tinged material as shown surrounding the membranous airways in Figure 3. There was increased collagen in both the small (Fig. 3C) and large airways (Fig. 3D) of the AdCMVAScftr-treated rats when compared to control airways of the same size (Fig. 3A &3B). Morphometric quantitation of airway collagen was performed using image analysis [20]. Airway collagen was increased significantly (p < 0.001) in the antisense treated animals as determined by an increased collagen/protein ratio (Table 1). Total lung collagen was also quantitated using image analysis [22]. As shown in Figure 4, statistically significant (p = 0.0029) increase in total collagen was confirmed.

**Figure 5:**
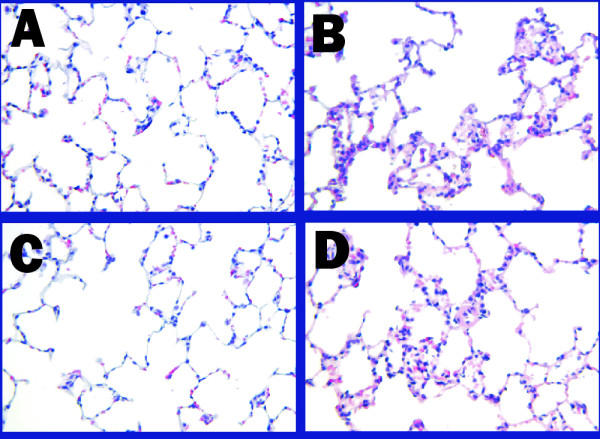
**Chronic inflammation following *in utero *AdCMVAScftr therapy**. Lung fields from rats at 100 days were examined for inflammation following hematoxylin and eosin staining (Panels A & B). Prominent areas of inflammatory cell infiltrate surrounding membranous airways were demonstrated in AdCMVAScftr treated rats (Panel B) and were not found in AdCMVgfp control animals (Panel A). Staining of the areas of inflammatory cell infiltrate with Masson's Trichrome demonstrated interstitial fibrosis associated with the inflammation (Panels C and D). Original magnification Panels A, B and C-100×; Panel D-400×.

Chronic inflammation is another feature of CF lung pathology in humans [23]. At 100 days of age, prominent inflammatory cell infiltrate was present in the lung parenchyma of the AdCMVAScftr treated rats (Fig. 5B &5D) that was not present in the AdCMVgfp-treated control animals (Fig. 5A &5C). We have previously demonstrated that *in utero *adenoviral-mediated transgene expression decreases rapidly in the 30 days post-transfer [2]. Thus, the adult rat lung pathology progressed in the absence of significant antisense *cftr *expression. In addition, although these animals were not kept in a germ free environment, repeated bacterial challenge was not required for the induction of either lung inflammation or fibrosis. All animals greater than 60 days of age thus far examined (n = 12) have had significant pulmonary inflammatory infiltrate.

### Expression of CF-specific proteins following TIUKO *cftr*

MRP8 and 14 are proteins previously used as clinical markers of cystic fibrosis. These proteins are calcium binding proteins that form a heterodimer, are produced in neutrophils, and are associated with wound healing. Importantly, MRP8 was originally called "CF antigen" because it was found to be elevated in the serum of CF patients. The protein was subsequently found to be a heterodimer of MRP8 and MRP14. It was used to identify CF affected individuals as well as heterozygous carriers prior to the discovery of the *cftr *gene. Because of their significance the expression levels of these proteins were confirmed by western blot analysis in both mice and rats following AdCMVAScftr gene therapy.

**Figure 6:**
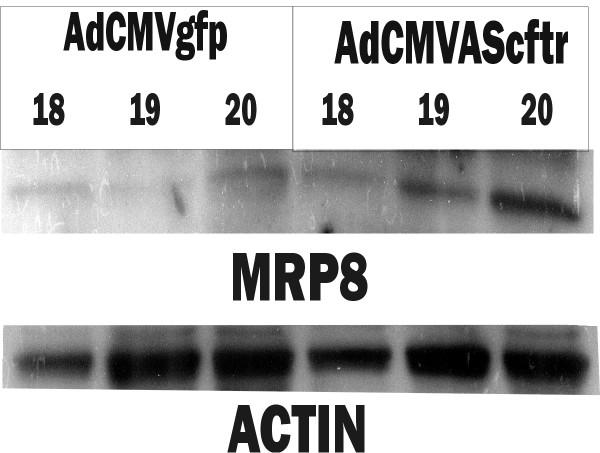
**Western analysis of MRP 8 (CF Antigen) expressions in rat lungs following *in utero *antisense *cftr *therapy**. Western blots were performed on protein (20 μg) from lungs using MRP8 or actin specific antibodies. Protein was extracted from either AdCMVgfp (control) or fetuses (n = 6) treated at 16 days gestation with AdCMVAScftr (n = 6) and fetal rat lungs harvested at 18–20 days gestation

To determine if MRP8/14 expression was directly associated with the inhibition of CFTR expression, and not induced by post-natal events, western blot analysis of expression was followed sequentially over the first 96 hours post antisense *cftr *therapy *in utero *. Minimally detected levels of MRP 8 were expressed in control fetuses (Fig. 6; AdCMVgfp-treated animals). In the AdCMVAS*cftr *-treated rats, a gradual increase in MRP 8 expression was documented over 96 hours post-therapy. Similar results were obtained with MRP 14 (data not shown). Thus, increased CF Antigen expression was correlated with the decreased expression of CFTR following antisense *cftr *gene therapy.

### Altered airway reactivity in antisense *cftr*-treated rats

**Figure 7:**
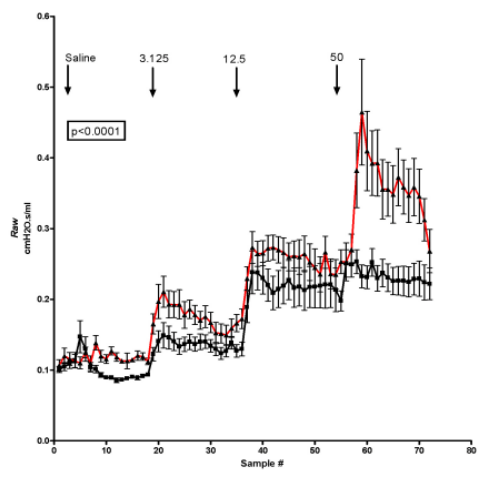
**Airway reactivity in AdCMVAScftr-treated rats**. Fetuses at 16 days gestation were treated with either AdCMVlacZ (black; n = 5) or AdCMVAScftr (red; n = 5). Animals were maintained in filtered cages to minimize exposure to environmental pathogens. At 6–12 months of age, changes in airway resistance (*Raw*) were determined in response to nebulized saline and acetylmethylcholine at concentrations of 3.125, 12.5, and 50 mg/ml.

In cystic fibrosis patients, alteration in airway reactivity was previously documented [24,25]. To evaluate the effect of *in utero *antisense *cftr *on the airway development rats treated at 16 days gestation with AdCMVAScftr or AdCMVlacZ were maintained in filtered cages and analyzed for airway reactivity to acetylcholine at 6–13 months of age. As shown in Fig. 7, control animals challenged with nebulized acetylcholine showed only small changes in airway resistance (*Raw*) at 3.125 and 12.5 mg/ml concentrations. In contrast, age-matched, antisense *cftr *treated animals were highly reactive to the low concentrations of acetylcholine. In addition, maximal stimulation at 50 mg/ml in the TIUKO CF rats was over twice that observed in control animals. The differences between control and TIUKO CF rats was highly significant (p < 0.0001)

## Discussion

The development of the TIUKO procedure permits the examination of mid-gestation developmentally required genes in the absence of both early lethality and compensatory mechanisms that ameliorate the final disease phenotype. Previously, transient expression of the antisense to a known growth factor *c-myc *[17] demonstrated its requirement for normal cell expansion in both the lungs and intestines. The key element in the TIUKO method is the targeting of multi-potential, undifferentiated cells. Because the lung is developing rapidly at the time, organogenesis can be dramatically affected by inhibition of genes involved in a developmental cascade. Thus, inhibition of *c-myc *gave rise to severely hypoplastic lungs and stunted villi formation in the intestines, even though fewer than 10^7 ^cells were affected by the antisense transgene. This method can be used to dissect the developmental pathway of different epithelial cell types in these organs.

One caveat with the TIUKO method, however, is the difficulty in measuring the decrease in expression of the target gene. Because the population of affected multipotential, undifferentiated cells represent a small proportion of the total, rapidly expanding lung population, it is impossible to detect the changes in gene expression via real time PCR, northern blots, or western blots. However, as shown previously two independent methods, antisense and ubiquitin targeted, down regulation of C-MYC [17] yielded identical phenotypes and immunofluorescent quantitated decrease of the target transgene,. In this paper, all conclusions are based only immunofluorescent quantitation of target gene down regulation following transient antisense CFTR *in utero *.

Cystic fibrosis is a pleiotropic disease. The seemingly unrelated phenotypic effects of CFTR are largely unexplained by the hypothesis that CF pathology results from the lack of continuous chloride channel expression. Beginning with the reversal of the CF knockout mouse phenotype with transient *in utero cftr *gene therapy using a recombinant adenovirus [9], this laboratory proposed that CF was also a disease of secretory cell differentiation and that the protein's many functions, including that of a chloride channel, were required for multipotential cell differentiation. Results supporting this hypothesis were obtained in mice, rats and non-human primates. Thus, some of the altered functions observed in CF tissues are due to incomplete development and malfunctioning secretory cells.

As shown recently [5], CFTR is highly expressed in the lung during the pseudoglandular phase of development and begins to decline during the cannalicular phase of development where it remains low at birth. This early phase of lung development correlates with that used for *in utero *gene therapy and reversal of the knockout mouse phenotype [5,13]. These data also suggested that the transient, selective, inhibition of CFTR expression should recapitulate the human CF phenotype without species-specific compensatory mechanisms interference. The development of the TIUKO method permitted such experiments.

The selective, transient CFTR expression inhibition in a small number (<10^7^) of undifferentiated multipotential cells was performed in using a recombinant adenovirus with a *cftr *fragment cloned in the 3'-5', antisense, direction. As shown in immunohistochemical examinations of lung tissues (Figure 1, 2), specific inhibition of CFTR expression occurred to the extent of that obtained previously with antisense *c-myc *[17].

In the lungs of TIUKO CF rats, significant changes in lung structure were not readily apparent at birth. As the animals aged, however, airway thickening and fibrosis were found morphologically. Changes in the airways were confirmed with morphometric analysis (Fig. 3, 4; Table 1) and pulmonary function tests (Fig 7). Thus, the TIUKO CF rats reproduced many aspects seen in lung disease of human.

Elevated serum levels of MRP8 were used to identify CF affected individuals and heterozygous carriers prior to the cloning of the *cftr *gene. The gene for the cystic fibrosis antigen (MRP8) was cloned in 1987 by Dorin et al. [20]. Because intermediate levels of the protein were expressed in clinically unaffected heterozygotes it was hypothesized at that time that its expression was closely related to the basic defect of cystic fibrosis. Work on this protein lost momentum when the *cftr *gene was cloned and confirmed to be a chloride channel and also when MRP 8 expression was not found in the preliminary survey of adult and fetal CF lung [26]. Because MRP8/14 is highly expressed in polymorphonuclear leukocytes, the high levels of MRP8/14 in CF patients were explained as a result of inflammation rather than a potential source of it. In addition to elevated levels of MRP8/14 protein in human CF serum, mRNA expression has been found in tracheal gland cells obtained from normal and cystic fibrosis patients. A significant increase in these mRNAs was shown in the cells of CF origin [27]. The increased expression of this protein in the fetal lung following antisense *cftr *gene transfer (Fig. 6) is consistent with human CF and the knockout mouse.

Reversal of the CF phenotype by *in utero *gene therapy and the developmental changes following CFTR over expression studies in mice, rats, and non-human primates are consistent with a developmental paradigm for this disease. As summarized in Table 2, the TIUKO CF rats demonstrate that faulty differentiation of secretory cell may be associated with many of the features of the CF lung disease phenotype [28].

**Table 2:** Comparison of cystic fibrosis disease phenotypes between human and animal models

**HUMAN DISEASE PHENOTYPE**	**CFTR KNOCKOUT MOUSE MODEL PHENOTYPE**	**TIUKO CF RAT PHENOTYPE**
LUNG FIBROSIS	FIBROSIS AND INFLAMMATION	PRESENT BY 100 DAYS OF AGE
MRP8/14 ELEVATED	DETECTED IN G551D MOUSE	INCREASED PRENATALLY
AIRWAY REACTIVITY	NOT DETECTED	INCREASED WITH AGE
CHRONIC INFLAMMATION	DETECTED IN CONGENIC MICE	PRESENT BY 100 DAYS OF AGE

Several recent papers illustrate the potential role of the developmental requirement of CFTR in CF pathophysiology and lung growth Groman and co-workers [29] found a subset of patients with the CF phenotype and no mutation in the *cftr *coding sequence. This finding is consistent with the role in CF of other genes in a common secretory cell pathway that includes *cftr *as only one of many components. In addition, transplant of human fetal CF lung tissues into SCID mice resulted in lung inflammation [30]. These data are consistent with our prenatal elevation of the MRP8/14 (Figure 6) and suggest that developmental interference with secretory cell differentiation results in a constitutive inflammatory response.

Until recently, no distinctive changes in lung structure and function were found in the CFTR knockout mouse. However, recent evaluation by our laboratory of lung function in *cftr+/+*, *cftr+/-*, and *cftr-/- *mice, showed distinct phenotypes for each genotype [31]. Thus, normal lung development in mouse is affected in a dose response manner by CFTR. The TIUKO rat is distinct genetically from a heterozygous animal. In heterozygous animals, one maintains a single functional copy of the transgene in all multipotential, undifferentiated cells of the developing fetal lung. So in the mature, heterozygous lung altered pulmonary function but normal structure is observed. In contrast, in the TIUKO CF fetal lung, multipotential, undifferentiated cells infected with the antisense gene have a total deficiency of CFTR (Fig 1). The developing TIUKO CF rat lung is a mosaic of normal (*cftr+/+*) and CFTR deficient (essentially *cftr-/-*). Thus, as shown in this paper, the TUIKO CF rat exhibited a CF-related phenotype while a CFTR heterozygous does not show any CF features.

**Figure 8:**
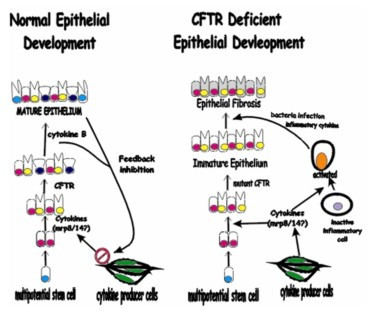
**Developmental Paradigm for Cystic Fibrosis Lung Fibrosis Based on TIUKO CF Rat and Developmental Studies in Mice, Rats, and Non-human Primates**. In Panel A CFTR is shown as one member of a developmental cascade required for normal secretory epithelium development. Included in this pathway are other cytokines, possibly MRP8/14. In normal development in the presence of CFTR feedback mechanisms either completely inhibit or at least decrease the expression of these developmentally active cytokines. In the absence of CFTR, Panel B, the secretory epithelium fails to differentiate properly. Failed development leads to an immature epithelium that does not exhibit the feedback function necessary for inhibition of developmentally required cytokines. Expression of these cytokines in the permanent, developmental immature, CF lung leads to activation of inflammatory cells once the immune system matures post-natal. Constitutive, chronic inflammation would explain the lung fibrosis and inflammatory disease seen in CF patients

We propose that CFTR is part of a developmental cascade for secretory cells in the lung, intestines, pancreas and other secretory organs (Fig. 8A). Disruption of this pathway could occur by either a *cftr *mutation, or as suggested by Groman et al's [29] work, other mutations of genes in this cascade. This would lead to incomplete differentiation of secretory cells and loss of function (Fig. 8B). In addition, the failure of secretory cell differentiation leads to a constitutive expression of cytokines that function in development as agents of differentiation. Once the immune system matures postnatally, however, these same cytokines assume a proinflammatory role, leading to chronic inflammation and fibrosis. The TIUKO CF rats may be used to identify these other genes involved in human lung epithelial cell differentiation and diseases resulting from their dysfunction. Finally, the TIUKO CF rat provides an animal model for the development of pharmacologic agents to disrupt the constitutional inflammatory processes in the CF affected tissues.

## Conclusions

Transient inhibition of CFTR expression in the lungs results in many features of cystic fibrosis in the mature animal. Increased fibrosis, chronic inflammation, increased airway reactivity, and elevation of CF antigen were observed. These data are consistent with a CFTR requirement for normal lung development.

## Methods

### Recombinant adenoviruses

A 920 bp human CFTR cDNA that included exons 1–6 (ATCC 61123; [18]) was gel-purified and subsequently subcloned into the plasmid pShuttle-CMV (Quantum Biotechnologies, Montreal, Canada). Recombinant adenoviruses were generated by homologous recombination in the *E. coli *strain BJ5183, according to the protocol of He et al [32]. Recombinants were confirmed for overall size by restriction endonuclease digestion and propagated in DH5a. Linear recombinant adenoviral DNA was used to transfect 911 packaging cells by Ca2PO4 precipitation to produce the virus AdCMVAScftr. Recombinant adenoviruses with the *lacZ *(AdCMVlacZ) and green fluorescent protein (GFP; AdCMVgfp) were provided by Dr. J. Kolls (LSHHSC, New Orleans, LA). All viruses were CsCl or HPLC purified.

### *In utero *gene transfer

Timed pregnant Sprague-Dawley Rats were induced (5%) and sedated (2%) with inhaled Isoflurane. A laparotomy was performed exposing the uterine horns. The individual amniotic sacs of the fetuses were visualized and injected with fine gauge needle containing adenoviral particles in 10% of the amniotic fluid volume. The recombinant adenoviruses in Dulbecco's Minimal Essential Medium delivered final concentrations of 10^8 ^pfu/ml to the amniotic fluid.

### Histochemistry and morphometry

At the time of sacrifice all animals received a number. This code was used for identification of all histologic and biochemical studies.

All tissues were fixed in methanol-free, 4% buffered paraformaldehyde and either mounted in paraffin or OCT for sectioning. Fluorescent immunohistochemistry was performed with goat polyclonal IgG (Santa Cruz) specific for CFTR carboxy (sc-8911) and amino terminal (sc-8909) sequences. Secondary donkey anti-goat ALEXA (Molecular Probes) antibodies were used. All tissues were visualized on a deconvoluting, Lieca, light microscope. Hematoxylin and eosin stain and Masson's Trichrome stain were performed with kits (Sigma Chemical Co) and tissues examined by standard light microscopy.

Morphometry was performed with the identification numbers and treatment groups unidentified by two blinded investigators. Airway thickness was determined following staining with hematoxylin and eosin in 100 day old rats. The area of the wall between the sub epithelial basement membrane and parenchymal epithelium was digitized excluding airway epithelium and cartilage. The corresponding segment of sub epithelial basement membrane was digitized and used as a reference length to normalize airway wall area [21]. Quantitation was performed using Scion Image [13,14].

Image analysis based morphometry was performed with the identification numbers and treatment groups unidentified. Morphometric analysis was performed by two blinded investigators. Digitalized images were analyzed for airway thickness using Scion Image [13,14] or for collagen using PHOTOSHOP imaging software [22,33]. Deconvoluting microscopy and image analysis was performed on a Lieca inverted microscope with Xenon light source and SLIDEBOOK imaging software.

### Western blots

Polyacrylamide gel electrophoresis was performed on 18% Tris-HCl gels (Biorad) and transferred to PVDF membranes (Amersham) [34,35]. Polyclonal antibodies to MRP8, MRP14, and actin (Santa Cruz) were used in the concentrations of 1:1000 (all antibodies). The secondary HRP-labeled anti-goat antibody (Santa Cruz) was incubated at a concentration of 1:8000. Detection was performed using ECL-plus (Amersham).

### Pulmonary function tests

Rats at 12–14 months of age were anesthetized with intra-peritoneal pentobarbital (90 mg/kg), and the trachea was dissected free of surrounding tissue and cannulated with a 20-gauge cannula. The rat was then connected to a small animal ventilator (*flexiVent*, SCIREQ Inc. Montreal, PQ, Canada) and ventilated with a tidal volume (V_t_) of 10 ml/kg; inspiratory:expiratory ratio (I:E) of 66.67%, respiratory rate of 150 breaths/minute, and maximum pressure of 30 cmH_2_0. Positive end-expiratory pressure (PEEP) was controlled by submerging the expiratory limb from the ventilator into a water trap. Each animal was paralyzed with pancuronium bromide (0.5 mg/kg) and allowed to equilibrate on the ventilator until spontaneous breathing ceased (5 minutes). *Zrs *measurements at a PEEP level of 3. Data were statistically evaluated using paired t-test.

#### Respiratory mechanics

To measure the input impedance of the respiratory system (*Zrs*), mechanical ventilation was interrupted and the animal was allowed to expire against the set level of PEEP for 1 s. We then applied an 8 second broad-band volume perturbation signal was then applied to the lungs with the *flexiVent*, after which ventilated was resumed. A PEEP of 3 cmH_2_O was used. The volume perturbation signal consisted of the superposition of 18 sine waves having frequency spaced roughly evenly over the range 0.25 Hz to 19.625 Hz. *Zrs *was calculated from the displacement of the ventilator's piston and the pressure in its cylinder as described previously [36,37]. Correction for gas compressibility as well as resistive and accelerative losses in the *flexiVent*, connecting tubing and the tracheal cannula were performed as described previously [38]using dynamic calibration data obtained by applying volume perturbations through the tubing and tracheal cannula first when it was completely closed and then when it was open to the atmosphere.

We interpreted the measurement of *Zrs *in terms of the constant phase model [39]



where *Raw *is a frequency independent Newtonian resistance reflecting that of the conducting airways [40], *Iaw *is airway gas inertance, *G *characterizes tissue damping, *H *characterizes tissue stiffness (elastance), *i *is the imaginary unit, *α *links *G *and *H*, and *f *is frequency. We also calculated a quantity known as hysteresivity (*η *= *G/H*), which is believed to increase when regional heterogeneities develop in the lung [41].

#### Acetylmethylcholine challenge

After rats were equilibrated on the respiratory, sequential 30 second challenges with nebulized physiologic saline, 3.125, 12.5 and 50 mg/ml acetylmethylcholine dissolved in physiologic saline were performed. Between each challenge, 18 broad-band volume perturbations were produced by the ventilator at 10 second intervals between each perturbation. *Raw *was calculated for each perturbation.

## Abbreviations

CFTR – Cystic fibrosis transmembrane conductance regulator; CF – cystic fibrosis

## Author contributions

Both authors were equally responsible for both the laboratory work and design of experiments
